# Reconstruction of a nail bed with double-layer artificial dermis for a pincer nail: A case report and literature review

**DOI:** 10.3389/fsurg.2022.1047171

**Published:** 2023-01-09

**Authors:** Weicong Deng, Zelin Zhou, Bo Leng, Xinming Zeng, Zi Shen, Yongchang Hong

**Affiliations:** ^1^ Department of Hand Microvascular Surgery, Affiliated Dongguan Hospital, Southern Medical University, Dongguan, China; ^2^ The First Clinical Medical School, The First Clinical Medical School of Guangzhou University of Chinese Medicine, Guangzhou, China

**Keywords:** pincer nail, artificial dermal, reconstitution, case report, literature review

## Abstract

**Background:**

A pincer nail (PN) is a type of nail deformity. Although it is a minor ailment, it can cause intractable pain, affecting daily work and life. Currently, there is no standard invention for PN. The main purpose of treatment is to correct the curvature of the nail, so we apply double-layer artificial dermis (DLAD), a novel treatment, for PN according to this aim.

**Case presentation:**

A 40-year-old man suffering from PN was treated with DLAD. After 1 year of follow-up, the patient's great toenail plate of the right foot was completely grown out. His pain was relieved, and the curvature of the toenail was corrected.

**Conclusion:**

The innovative method of using DLAD to fill the ingrown nail bed not only solves the problem of recurrent pincer nail deformity but also restores the original appearance of the toenail. It suggested that this simple procedure can be widely applied in clinical practice.

## Introduction

A pincer nail (PN) was first named a sunken nail by Frost (1) in 1950. It is characterized by the thickening of the nail, progressive narrowing of the nail bed from near to far along the longitudinal axis, and increasing lateral nail curvature. As the deformity worsens, the patient gradually develops pain around the toenail, chronic inflammation, and recurrent infections. Patients with severe symptoms are unable to wear shoes and walk, and some even choose to amputate the toe to relieve the pain. As a new material for regenerative repair of trauma, the double-layer artificial dermis (DLAD) has good penetration and tissue regeneration ability, which is effective in repairing tissue defects. We have innovatively used DLAD to fill the nail bed to treat a patient's PN and report it currently. The patient has given informed consent for the publication of this case report.

## Case presentation

**Figure 1:**
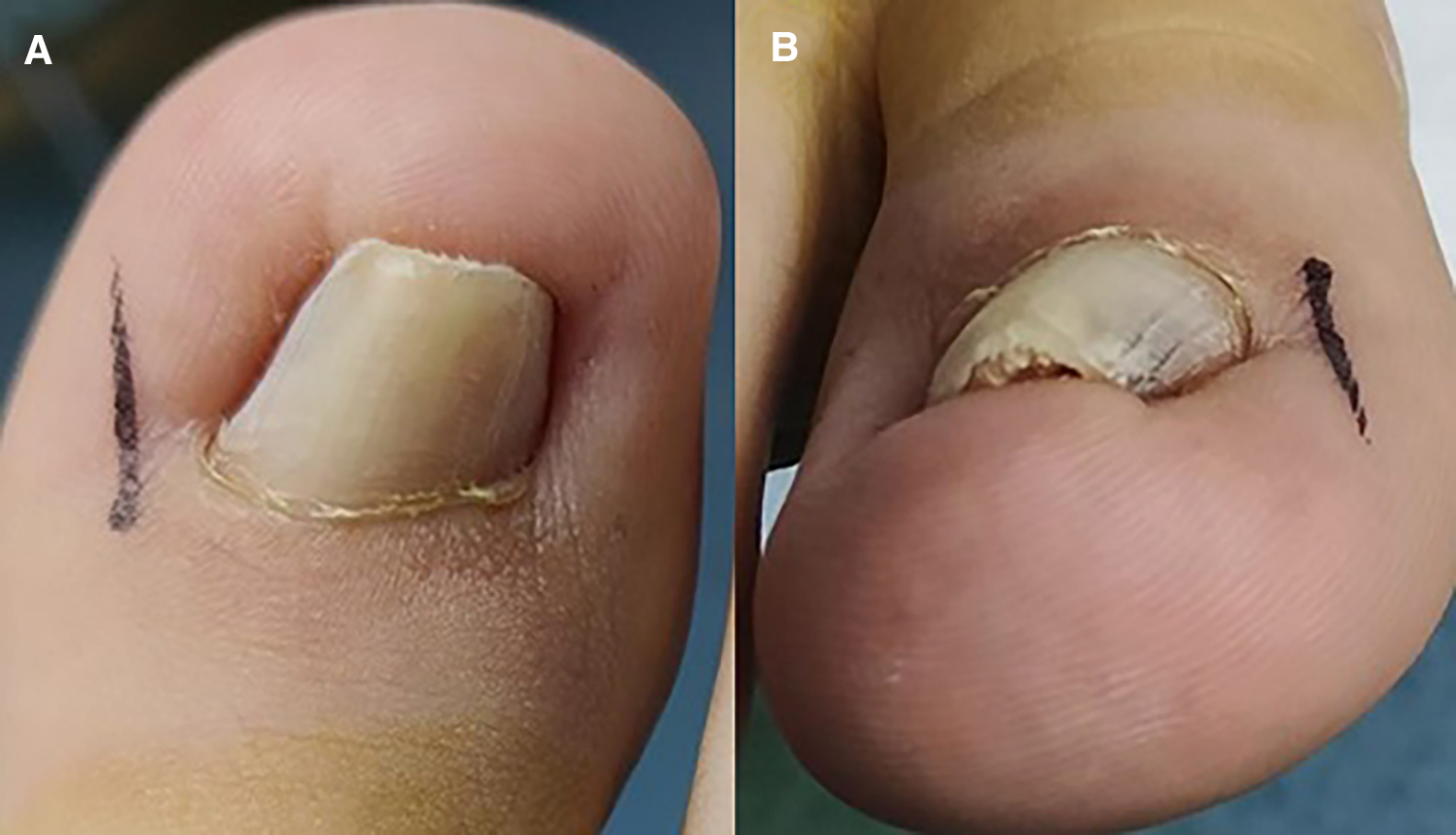
Preoperative appearance of affected great toenails at different angles (**A,B**).

A 40-year-old man presented with painful inflammation of the right toenail for 3 years. He had undergone several nail extractions and long-term antibiotic treatment. However, the pincer nail continued to recur after several surgeries. The patient had no relevant family history of genetic disorders and the absence of skin diseases such as nail moss on the right foot. Physical examination revealed a pincer-like deformity (Figures 1A,B) of the right great toenail with peri-nail redness and swelling without pus exudation. The rest of the toes did not show pincer deformity. Measurement using a method proposed by Yabe (2) in 2013 to assess the severity of the PN revealed a curvature index of 64.5%. It belonged to type 1 according to the Baran classification (3). At the same time, the patient was evaluated with a VAS score of 6.

### Procedure

**Figure 2:**
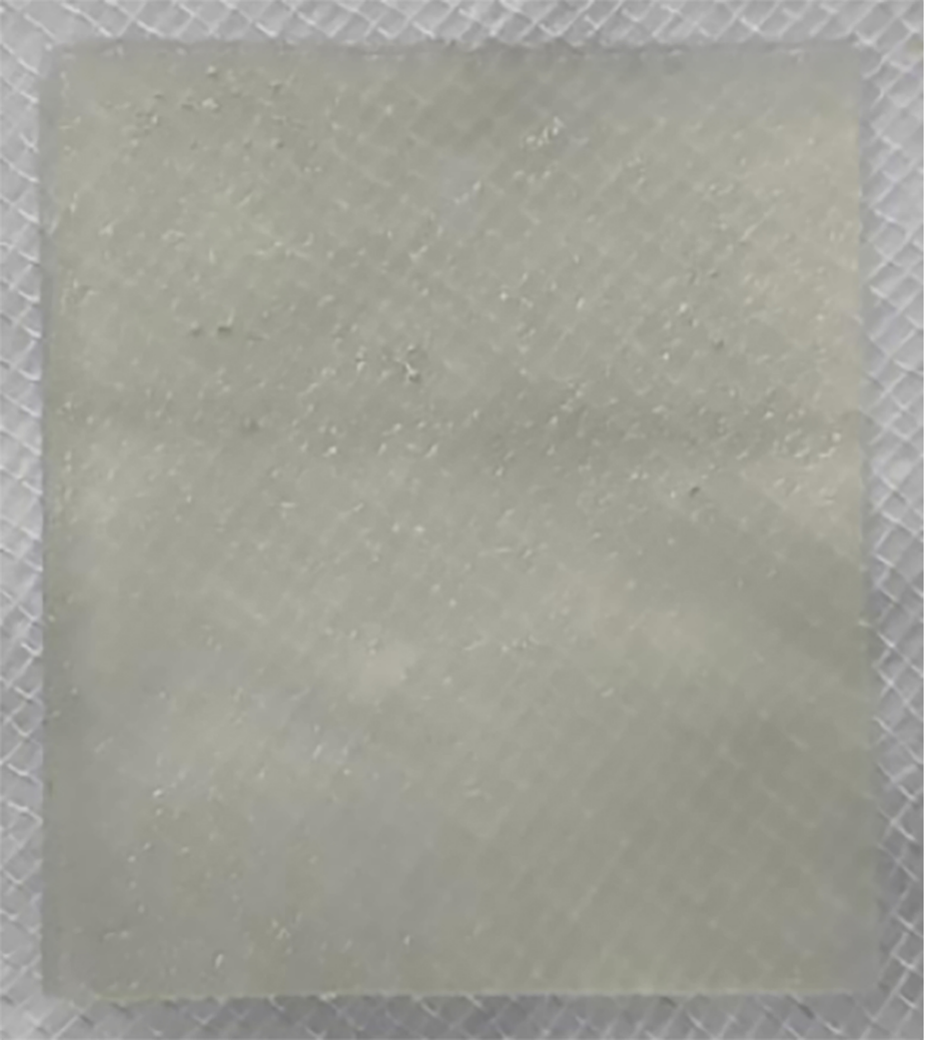
Appearance of double-layer artificial dermis.

**Figure 3:**
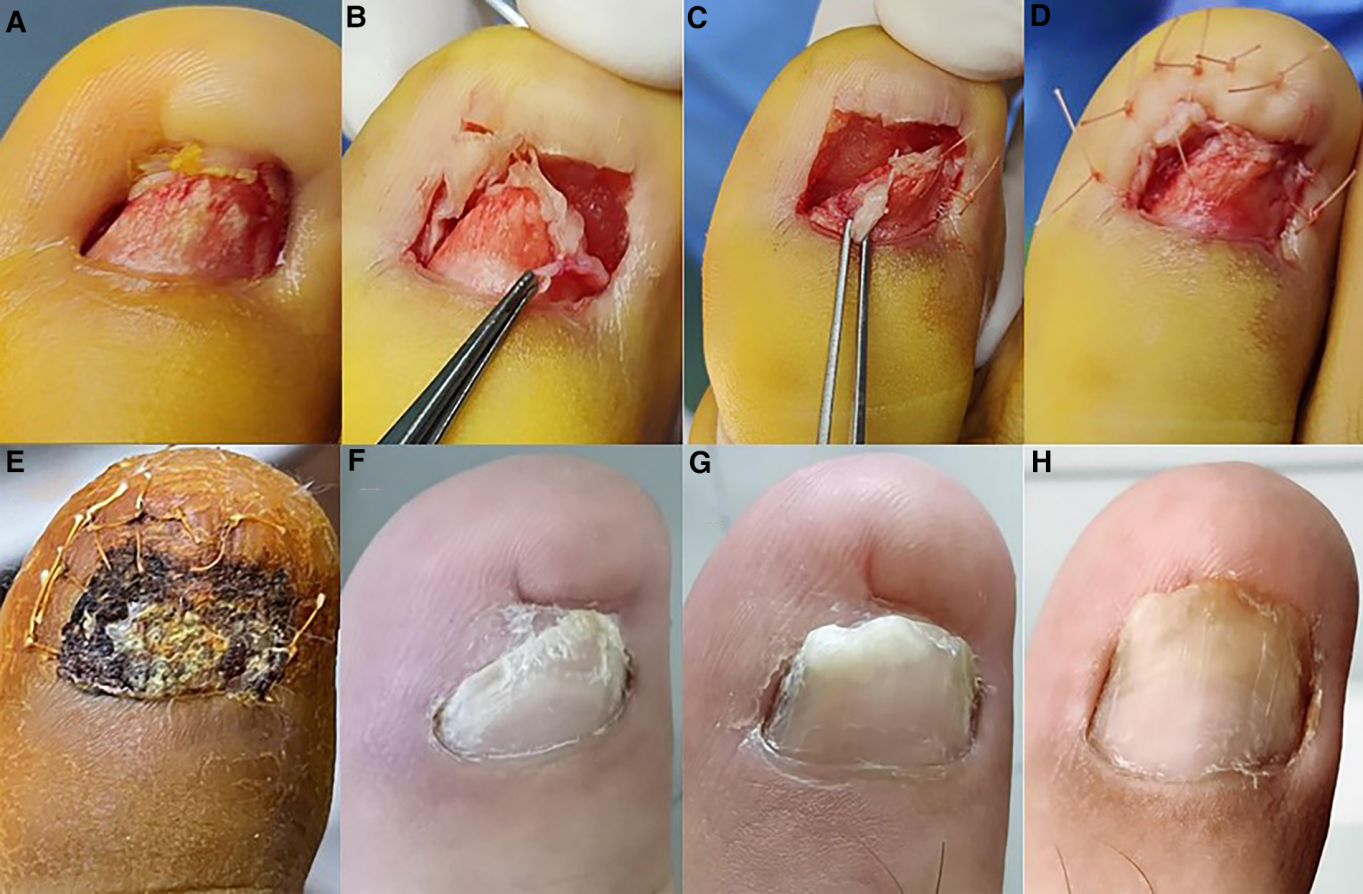
Toenail bed was embedded before surgery (**A**). Artificial dermis was filled on both sides of the affected toe (**B**). After the artificial dermis filling, the nail bed was obviously smooth (**C**). Postoperative appearance of the affected toe (**D**). Appearance of the affected toe 1 month after surgery (**E**). Appearance of the affected toe 3 months after surgery (**F**). Appearance of the affected toe 9 months after surgery (**G**). Appearance of the affected toe 1 year after surgery (**H**).

**Figure 4:**
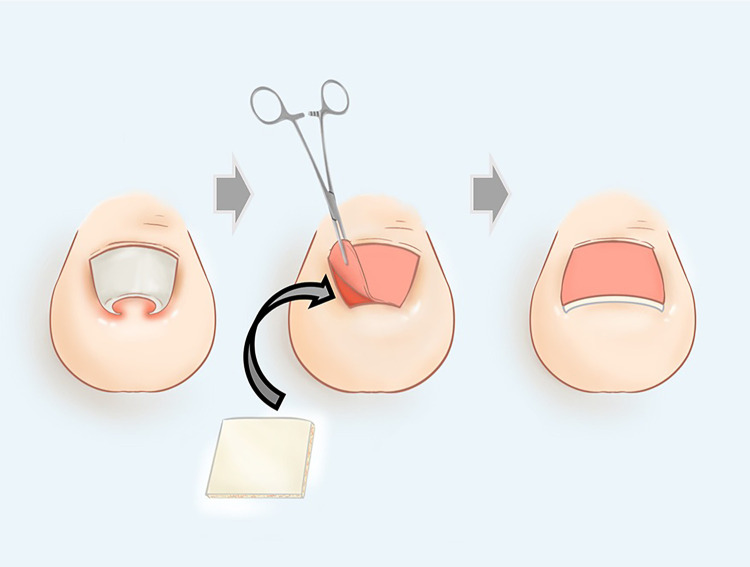
Schematic diagram of the procedure.

The patient underwent the procedure under combined spinal and epidural anesthesia with tourniquet control. First, the ingrown nail plate was turned up and removed with mosquito forceps. When removing the nail, we need to protect the nail root, which has the nail matrix. Proliferation, keratinization, and forward migration of the nail cells determine the growth of the toenail. We then trimmed the edges of the nail bed and separated the nail bed from the lateral nail fold by cutting through the border of both sides of the embedded segment. We suggest the incision be at least 3-mm-long from the nail root. Too long an incision will affect the nutrient supply to the nail bed, while too short an incision will not adequately correct the ingrown nail. Subsequently, the nail bed was dissected from the phalanx with a scalpel at the edge of the incised nail bed along the nail grooves bilaterally. We made the separation to preserve the nail midline at least 5 mm wide to minimize the disruption of blood circulation to the nail bed. Be careful to maintain the integrity of the nail bed. We turned over the nail bed to expose the sub-bed tissue. After that, the DLAD (Figure 2) from Lando, Shenzhen was soaked in saline for 5 min, and the surface silicone layer was removed. The remaining multinull matrix complex composed of depsipeptide-activated collagen and chondroitin sulfate was filled with the subnail bed tissue. There needs to be sufficient filling below the nail bed in both inset nail grooves to bring the nail bed closer to the physiological curvature. It is also vital to ensure that the proper width of the nail groove is retained to allow for nail plate growth. Finally, the nail bed was re-covered back onto the toe bone and checked again to see whether the nail bed was level. The shape of the nail bed determines the shape of the new toenail, and if the nail bed is still ingrown, the pincer nail may recur. Next, the nail bed was resutured with its original corresponding lateral nail crease using 4-0 absorbable sutures to form a new nail groove (avoiding dislodgement of the filled multivessel matrix complex). Finally, we covered the surface with oiled gauze and bandaged the surgical incision. The whole operation process is shown in Figures 3A–D. After the procedure was completed, antibiotics and anti-inflammatory pain management were given to the patient. The schematic diagram of this procedure is shown in Figure 4.

### Follow-up

The dressing was replaced every 1–2 days postoperatively, and the patient was told to wear loose shoes postoperatively. The follow-up 1 month after surgery showed complete healing of the surgical wound and well growth of the nail with the DLAD (Figure 3E). About 3 mm of nail plate growth could be observed at this time. Then, at the 3-month postoperative follow-up, about 15 mm of nail plate growth was seen. The growth rate of the bilateral area of the nail was lower than that of the middle part of the nail, and the nail plate was abnormally thickened (Figure 3F). Probably because of the damage to the nail grooves on both sides of the affected toe after repeated nail extractions. Nine months after the operation, we saw that the nail plate growth improved obviously, the curvature index reached 87.5%, and the VAS score reduced to 1 (Figure 3G). So far, the nail plate is fully grown and looks good; the patient has not felt pain and is back to work, feeling satisfied with this operation (Figure 3H).

## Discussion

PN is a rare disorder with an incidence of approximately 0.9%, much lower than the 7.4% incidence of ingrown toenails. It occurs on one or both sides of the toenail (4). The cause of PN is unknown, and the prevailing view is that it is either hereditary or acquired. Chapman has reported three generations of hereditary PN, concluding that the disorder is autosomal-dominant and often presents as symmetrical (5). In addition, Hu et al. (6) illustrated a case of a multigenerational Taiwanese family in which about half of the family members had PN, which also confirms the possibility that the occurrence of PN is inherited. Unlike hereditary PN, acquired PN often presents asymmetrically, and its prevalence is associated with inappropriate shoes, developmental abnormalities, nail moss, beta-blocker use, and some systemic diseases (renal failure, systemic lupus erythematosus, gastrointestinal malignancies) (7–9). For acquired PN, improvement of lifestyle habits, discontinuation of suspected drugs, and treatment of the primary disease can improve the symptoms to some extent.

PN and ingrown nails are both toe diseases and share many similarities, leading to many clinical misdiagnoses. Visually, the major difference between the two is that the shape of the nail in ingrown toenails is basically normal. In PN, the width of the nail gradually decreases from the root of the nail, and the distal nail plate even appears to curl inward, while the height of the nail gradually increases and the nail plate appears to be abnormally thickened. This is a significant difference from ingrown nails and should be distinguished clinically (10).

For PN, surgical treatment is more varied, including simple nail extraction, partial nail mass excision, and nail bed plasty. Suzuki et al. (11) treated PN by increasing the area of the nail bed with a severed skin graft, but according to follow-up, the toenail eventually failed to adhere to the nail bed and fell off. Mutaf et al. (12) enlarged the distal part of the nail bed laterally by the 5-flap Z plasty method, thus changing the appearance of the nail bed. However, this procedure is more complicated and more damaging to the nail bed and is not used much in clinical practice. Shin et al. (13) treated PN by occluding the protruding bone of the distal phalanx, burning the proximal ingrown nail bed with electrodes, and smoothing the nail plate; this method eliminated the excess bone under the nail bed. The burning of the ingrown nail bed also prevented the nail plate from forming an ingrown nail due to the lack of a “platform,” preventing bone destruction and skin necrosis. In addition, Leshin and Whitaker (14) described permanent nail ablation with the CO_2_ laser, but this treatment required specific instrumentation and was not compatible with widespread dissemination.

The DLAD, a new synthetic material, has been widely used in plastic surgery and dermatology. Its upper layer is a semipermeable silicone membrane, and the lower layer is a porous matrix composed of deuterated peptide bovine Achilles tendon collagen and glycosaminoglycan. It provides a stable scaffold for tissue growth and promotes the trabecular capillaries and fibroblasts from the trabecular base and surrounding tissues into it. A scaffold-new capillary-cell complex appeared, and the material will gradually degrade with new tissue that will substitute.

To correct the curvature of the nail, we start by correcting the curvature of the nail bed by preserving the nail matrix. Based on the good penetration and scaffolding effect of the DLAD, we innovatively used the DLAD to fill into the nail matrix, thus achieving the purpose of nail bed flattening. The results of this procedure are satisfactory for treating PN through follow-up. The advantage of this procedure is that it is simple to perform, as only the nail bed is separated during the procedure and the DLAD is subsequently inserted. The procedure can be performed with simple training. Furthermore, this procedure protects the integrity of the nail bed to the greatest extent possible. Due to excessive destruction of the nail bed, abnormal nail growth will occur, such as nail fractures and abnormal thickening of the nail plate. In our approach, the nail bed is separated intact without any trimming of the nail bed and finally the nail bed is sutured to both walls without tension. There is no disruption of the nail bed, and therefore, no abnormal nail growth has occurred in patients who have undergone this procedure. At the same time, this procedure corrects the curvature of the nail to a great extent by filling in the nail bed, and the new nail plate follows the flat nail bed and does not reappear as PN. We believe that the recurrence rate of PN will be greatly reduced by this method.

Despite the many advantages, we found a problem that is worth considering. We left a wide enough nail groove, but the DLAD grows faster than the nail plate, which tends to heal too quickly and adhere to the nail folds on both sides. As a result, the nail sulcus was closed, so the nail plate lacked a “track” to slide forward, preventing the growth of the nail plate.

## Conclusion

With our presented case, we validated DLAD improves healing with significant efficacy. However, some technical problems still need to be solved. Therefore, we need more clinical evidence to support the efficacy of this procedure and to improve it in the future.

## Data Availability

The original contributions presented in the study are included in the article/Supplementary Material, further inquiries can be directed to the corresponding author.
